# Clathrin Heavy Chain 1 Plays Essential Roles During Oocyte Meiotic Spindle Formation and Early Embryonic Development in Sheep

**DOI:** 10.3389/fcell.2021.609311

**Published:** 2021-02-25

**Authors:** Zhe Han, Xin Hao, Cheng-Jie Zhou, Jun Wang, Xin Wen, Xing-Yue Wang, De-Jian Zhang, Cheng-Guang Liang

**Affiliations:** State Key Laboratory of Reproductive Regulation and Breeding of Grassland Livestock, School of Life Sciences, Inner Mongolia University, Hohhot, China

**Keywords:** CLTC, spindle assembly, chromosome congression, oocyte, early embryo development

## Abstract

As a major protein of the polyhedral coat of coated pits and vesicles, clathrin molecules have been shown to play a stabilization role for kinetochore fibers of the mitotic spindle by acting as inter-microtubule bridges. Clathrin heavy chain 1 (CLTC), the basic subunit of the clathrin coat, plays vital roles in both spindle assembly and chromosome congression during somatic-cell mitosis. However, its function in oocyte meiotic maturation and early embryo development in mammals, especially in domesticated animals, has not been fully investigated. In this study, the expression profiles and functional roles of CLTC in sheep oocytes were investigated. Our results showed that the expression of CLTC was maintained at a high level from the germinal vesicle (GV) stage to metaphase II stage and that CLTC was distributed diffusely in the cytoplasm of cells at interphase, from the GV stage to the blastocyst stage. After GV breakdown (GVBD), CLTC co-localized with beta-tubulin during metaphase. Oocyte treatments with taxol, nocodazole, or cold did not affect CLTC expression levels but led to disorders of its distribution. Functional impairment of CLTC by specific morpholino injections in GV-stage oocytes led to disruptions in spindle assembly and chromosomal alignment, accompanied by impaired first polar body (PB1) emissions. In addition, knockdown of CLTC before parthenogenetic activation disrupted spindle formation and impaired early embryo development. Taken together, the results demonstrate that CLTC plays a vital role in sheep oocyte maturation via the regulation of spindle dynamics and an essential role during early embryo development.

## Introduction

Successful oocyte maturation and early embryo development depend on correct spindle formation and chromosome separation during cell division (Duan and Sun, 2019; Radonova et al., 2019). Similar to somatic cells, the oocyte meiotic spindle is mainly composed of microtubules (MTs), and spindle organization depends on the activities of numerous MT-associated proteins (Xu et al., 2012; Petry, 2016; Namgoong and Kim, 2018). Well-organized spindles are responsible for maintaining correct chromosome alignment and separation during meiosis I and meiosis II (Dumont and Desai, 2012). Conversely, most oocyte aneuploidy is caused by chromosome segregation errors during meiotic divisions, leading to embryo development failures or neonatal defects (Webster and Schuh, 2017; Mogessie et al., 2018).

Clathrin, a major protein component of the cytoplasmic face of intracellular organelles, is composed of three heavy chains and three light chains (Kirchhausen and Harrison, 1981; Fotin et al., 2004; Royle and Lagnado, 2006). During interphase, clathrin is involved in the intracellular trafficking of receptors and the endocytosis of a variety of macromolecules (Kirchhausen, 2000; Fielding and Royle, 2013; He et al., 2017). During mitosis, clathrin molecules stabilize kinetochore fibers of the mitotic spindle by acting as inter-MT bridges and promote chromosome congression in somatic cells (Booth et al., 2011; Cheeseman et al., 2011; Rao et al., 2016). Clathrin has been shown to interact directly with the spindle through the N-terminal domain of clathrin heavy chain 1 (CLTC) (Royle and Lagnado, 2006). Through the recruitment of the phosphorylated form of transforming acidic coiled coil 3 (TACC3) and colonic and hepatic tumor overexpressed gene (ch-TOG), CLTC forms a complex known as an inter-MT bridge (Fu et al., 2010; Booth et al., 2011), which has been shown to stabilize the MTs of the mitotic spindle (Lin et al., 2010). In addition, CLTC has been shown to interact with sorting nexin 9 (SNX9) to stabilize the kinetochore fibers of the mitotic spindle (Ma et al., 2013). Centrosome positioning in *Caenorhabditis elegans* embryos has also been shown to be regulated by CLTC (Spiro et al., 2014).

For meiosis, meiotic spindle-associated clathrin was initially shown in mouse metaphase II (MII) oocytes (Maro et al., 1985). Our previous mouse oocyte studies have shown that CLTC plays important roles in both spindle assembly and chromosome congression by interacting with cytoskeleton-associated protein 5 (CKAP5) (Han et al., 2010; Zhao et al., 2013; Lu et al., 2017). In porcine oocytes, clathrin has been shown to be crucial for stabilizing the metaphase spindle (Holzenspies et al., 2010). Although roles for CLTC in oocyte spindle formation and chromosome congression have been well studied in both mouse and porcine models, it remains unclear if CLTC affects oocyte maturation and embryo development via similar mechanisms in artiodactyl animals such as sheep, as significant differences exist between artiodactyl and rodent oocytes. For example, sheep oocytes are larger (∼120 μm in diameter) than mouse oocytes (∼70–80 μm in diameter) (Kim et al., 2004; Cotterill et al., 2013). In sheep, oocyte maturation time (from follicle release to stage MII) requires 22–24 h, much longer than that required for mouse oocytes (Wang et al., 2018, 2019). This longer maturation process in sheep oocytes also requires more MT stability to regulate chromosomal separations and may be affected by the existence of an undefined microtubular network observed in some goat MII oocytes (Velilla et al., 2005). Therefore, further investigations are warranted to determine if sheep and mouse oocytes share the same CLTC-regulated mechanisms for spindle formation and chromosome separation. Here, to further fundamental knowledge of domesticated animal reproduction, we report the expression and function of CLTC during sheep oocyte maturation and early embryo development.

## Materials and Methods

### Ethics Statement

All study procedures were performed according to the National Research Council Guide for the Care and Use of Laboratory Animals and were approved by the Ethics Committee of Inner Mongolia University (Approval number: SYXK 2014-0002).

### Cumulus–Oocyte Complex Collection and *in vitro* Maturation

Sheep ovaries were obtained from a local slaughterhouse and transported to the laboratory in saline solution (0.9% w/v) at 37°C within 2 h after slaughter. Mature follicles (2- to 6-mm diameters) were cut to release the cumulus–oocyte complexes (COCs) into cell culture dishes (Biosharp, Beijing, China) containing pre-warmed HEPES-buffered TCM-199 (11150059, Thermo Fisher Scientific, Waltham, MA, United States) supplemented with 50 IU/ml of heparin (H32020612, Wanbang Biopharma, Jiangsu, China). Only COCs with intact cumulus cell masses were selected for *in vitro* maturation (IVM). COCs were washed and cultured in oocyte maturation medium [TCM-199 supplemented with 0.02 IU/ml of follicle-stimulating hormone (FSH; F2293, Sigma-Aldrich, St. Louis, MO, United States), 1.0 IU/ml of luteinizing hormone (LH; L6420, Sigma-Aldrich), 0.1 μg/ml of β-estradiol (E2, E2758, Sigma-Aldrich), 0.22 mg/ml of sodium pyruvate (P4562, Sigma-Aldrich), 10% fetal bovine serum (FBS; 16000-044, Thermo Fisher Scientific), and 1% penicillin–streptomycin (15140122, Thermo Fisher Scientific)]. Fully grown germinal vesicle (GV)-stage oocytes were cultured in a humidified atmosphere of 5% CO_2_ at 38.5°C for 24 h.

### Parthenogenetic Activation and *in vitro* Embryo Development

Cumulus cells from matured COCs were peeled off by gentle pipetting in HEPES-buffered TCM-199 supplemented with 0.3 mg/ml of hyaluronidase (H3506, Sigma-Aldrich). Subsequently, denuded oocytes were activated using a potent Ca^2+^ ionophore (5 μM of ionomycin, C7522, Sigma-Aldrich) for 5 min and rapidly transferred to culture medium containing 2 mM of 6-(dimethylamino) purine (6-DMAP; D2629, Sigma-Aldrich) for 3.5 h. After activation, the parthenogenetic embryos were washed three times and transferred into synthetic oviduct fluid medium [119.2 mM of NaCl (S5886, Sigma-Aldrich), 7.9 mM of KCl (P5405, Sigma-Aldrich), 1.3 mM of KH_2_PO_4_ (P5655, Sigma-Aldrich), 0.8 mM of MgSO_4_⋅7H_2_O (M1880, Sigma-Aldrich), 0.66 μl/ml of Na-lactate (L7900, Sigma-Aldrich), 25.0 mM of NaHCO_3_ (S5761, Sigma-Aldrich), 0.7 mM of sodium pyruvate, and 1.8 mM of CaCl_2_⋅2H_2_O (C7902, Sigma-Aldrich)] supplemented with 29.2 μg/ml of L-glutamine (G8540, Sigma-Aldrich), 0.5 mg/ml of inositol (I7508, Sigma-Aldrich), 6 mg/ml of bovine serum albumin (BSA; A3311, Sigma-Aldrich), 30 μl/ml of essential amino acid (B6766, Sigma-Aldrich), and 10 μl/ml of MEM non-essential amino acid solution (M7145, Sigma-Aldrich). Embryos were cultured at 38.5°C in a humidified atmosphere of 5% CO_2_ for 48 h. The embryos were then transferred and cultured for 5 days in synthetic oviduct fluid medium supplemented with 29.2 μg/ml of L-glutamine, 0.5 mg/ml inositol, 4% FBS, 30 μl/ml of essential amino acid, and 10 μl/ml of MEM non-essential amino acid solution. Embryos were assessed for developmental stages at days 1, 2, 3, 4, and 5 to calculate two-cell, four-cell, morula, and blastocyst percentages, respectively. For the developmental rates for all embryos, the number of MII oocytes subjected to parthenogenetic activation was used as the denominator.

### Immunofluorescence

Oocytes or embryos were fixed in 4% paraformaldehyde (157-8, Electron Microscopy Sciences, Hatfield, PA, United States) for 30 min at room temperature. Samples were permeabilized with 1% Triton X-100 (T8532, Sigma-Aldrich) overnight at 37°C and blocked with 2% BSA for 1 h at room temperature. Next, the samples were incubated with primary antibodies against CLTC (rabbit polyclonal, 1:1,000, ab21679, Abcam, Cambridge, United Kingdom) and beta-tubulin (mouse monoclonal, 1:2,000, ab44928, Abcam) overnight at 4°C. The samples were then incubated with rhodamine-conjugated donkey anti-rabbit IgG (1:300, 711-025-152, Jackson ImmunoResearch Laboratories, West Grove, PA, United States) or Dylight^TM^ 488-conjugated AffiniPure donkey anti-mouse IgG (1:800, 715-485-150, Jackson ImmunoResearch Laboratories) for 1 h at 37°C. DNA was stained using 5 μg/ml of 4′,6-diamidino-2-phenylindole (DAPI; 236276, Roche, Mannheim, German) at room temperature. Finally, specimens were mounted on glass slides and observed using a confocal laser scanning microscope (A1R, Nikon, Tokyo, Japan).

### Immunoblotting

Immunoblotting was conducted as previously described (Zhou et al., 2019). Briefly, a rabbit polyclonal anti-CLTC (1:1,000, Abcam) or a mouse monoclonal anti-beta-actin (1:100, sc-69879, Santa Cruz Biotechnology, Dallas, TX, United States) was used as a primary antibody. Bands on membranes were detected using an Enhanced Chemiluminescence Detection Kit (32106, Thermo Fisher Scientific), and images were captured using a Tanon-5200 imaging system (Tanon, Shanghai, China).

### Drug Treatments

For taxol treatment, 5 mM of taxol (T7191, Sigma-Aldrich) stock in dimethyl sulfoxide (DMSO; D5879, Sigma-Aldrich) solution was diluted with TCM-199 to a final concentration of 25 μM. Oocytes at metaphase I (MI) and MII stages were treated with taxol for 80 min and then fixed for immunostaining or collected for immunoblotting. The control group contained DMSO at the same concentration as the 25 μM of taxol treatment group. For nocodazole treatment, 10 mg/ml of nocodazole (M1404, Sigma-Aldrich) in DMSO solution was diluted with TCM-199 to a final concentration of 20 μg/ml. Oocytes at MI and MII stages were treated with nocodazole for 50 min and then collected for immunostaining or immunoblotting. The control group contained DMSO at the same concentration as the 20 μg/ml nocodazole treatment group.

### Cold-Mediated Microtubule Depolymerization Assay

A cold-mediated MT depolymerization assay was performed by placing oocytes in oocyte maturation medium at 4°C for 1 h. Some oocytes were randomly selected and immediately collected for immunostaining or immunoblotting. Others were placed in oocyte maturation medium at 38.5°C in a humidified atmosphere of 5% CO_2_ for 30 min as a rescue treatment and then collected for immunostaining or immunoblotting.

### Morpholino Microinjections

Based on the sequence of CLTC in sheep (Ensembl: ENSOARG00000013249), *CLTC* morpholino (MO) (5′-TCT GAC TCC ATA GTC AAG GTA CTG A-3′) was designed and synthesized (Gene Tools, Philomath, OR, United States). Ten-picoliter volumes of either *CLTC*-MO or negative control-MO (5′-CCT CTT ACC TCA GTT ACA ATT TAT A-3′), both at concentrations of 2 mM, were microinjected into the cytoplasm of GV-stage oocytes in HEPES-buffered TCM-199 supplemented with 10 μM of milrinone (M4659, Sigma-Aldrich). After the microinjections, the oocytes were incubated for 20 h in oocyte maturation medium with 10 μM of milrinone to allow sufficient time for MO blocking of maternal mRNA. The oocytes were then washed three times and cultured for 24 h in oocyte maturation medium without milrinone to facilitate oocyte maturation.

For the CLTC translational block experiments in sheep embryos, 10 pl of 2 mM *CLTC*-MO or control-MO was injected into mature oocytes. After the microinjections, the oocytes were incubated for 30 min in oocyte maturation medium and then subjected to parthenogenetic activation.

### Statistical Analyses

Data are shown as means ± standard deviations (SD) from at least three replicates of each experiment. Statistical comparisons were made using two-tailed Student’s *t*-tests for absolute values and were calculated using Microsoft Excel software. The percentages for early embryo development (two-cell, four-cell, eight-cell, morula, and blastocyst stages) were compared using chi-squared tests (Microsoft Excel software). *P* < 0.05 values were considered statistically significant.

## Results

### Expression and Subcellular Localization of Clathrin Heavy Chain 1 During Sheep Oocyte Meiotic Maturation and Early Embryo Development

**FIGURE 1:**
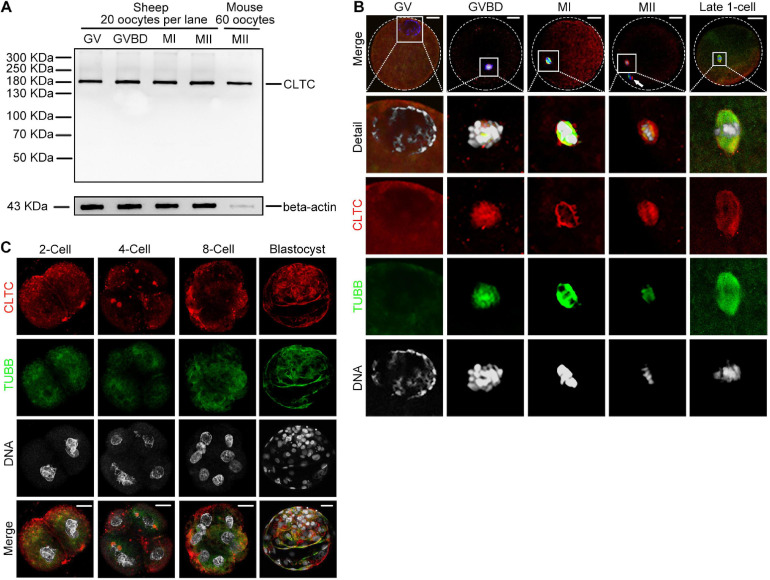
Expression and subcellular localization of CLTC during sheep oocyte meiotic maturation and early embryonic development. **(A)** Immunoblotting analysis of CLTC in sheep oocytes during different stages of maturation (GV, GVBD, MI, and MII) and in mouse oocytes at MII stage. Lysate from 20 sheep oocytes or 60 mouse oocytes was loaded in the corresponding lanes. Beta-actin was used as a loading control. Oocyte samples were collected after being cultured for 0, 8, 12, and 24 h to reach the stages of GV, GVBD, MI, and MII, respectively. **(B,C)** Subcellular localizations of CLTC at each stage of oocyte and early embryonic development were detected by immunofluorescence staining. Slides were examined using a confocal microscope. White arrow indicates a polar body. Red, CLTC; green, TUBB; blue, DNA. Scale bar = 20 μm. CLTC, clathrin heavy chain 1; GV, germinal vesicle; GVBD, germinal vesicle breakdown; MI, metaphase I; MII, metaphase II.

Clathrin heavy chain 1 protein expression and subcellular localization during sheep oocyte meiotic maturation were examined by immunoblotting and immunofluorescence staining, respectively. The CLTC antibody used in our previous mouse study was also used in the current study. However, whether this antibody is specific and sensitive enough to label CLTC in sheep oocytes has not yet been demonstrated. To validate this antibody in sheep oocytes, sheep and mouse oocyte lysates were loaded onto the same gel to compare labeled bands. Lysate from 20 sheep oocytes or 60 mouse oocytes were loaded in the corresponding lanes. We found that the single bands from both sheep and mouse oocyte extracts had the same molecular weight, and we matched the molecular weight (∼180 kDa) of the corresponding antigen. The immunoblotting analysis showed that CLTC was highly expressed in the GV, GV breakdown (GVBD), MI, and MII stages. Due to the bigger size of the oocytes in sheep, higher levels of beta-actin were expressed in sheep oocytes than that in mice (Figure 1A). We also determined that CLTC in mouse and sheep shared 99.8% protein sequence identity (data not shown). Combined with the immunoblotting results that CLTC was expressed in GV-stage oocytes (Figure 1A), this suggests that CLTC was distributed evenly throughout the GV stage cytoplasm. After the GVBD stage, CLTC was found to be concentrated around the chromosomes in the spindle area and co-localized with tubulin at the MI and MII stages (Figure 1B). Briefly, CLTC may be functionally associated with tubulin during sheep oocyte meiosis. After parthenogenetic activation, embryos developed to late one-cell, two-cell, four-cell, eight-cell, and blastocyst stages. Again, CLTC redistributed evenly within the cytoplasm during interphase for each embryo stages. However, focal accumulations of CLTC in both two-cell and four-cell embryos (in addition to diffuse cytoplasmic staining) were also observed. Similar to its localization in MII-stage oocytes, accumulations of CLTC in the mitotic spindles of metaphase one-cell-stage embryos were also observed, illustrating a similar potential role for CLTC in both meiosis and mitosis (Figures 1B,C).

### Microtubule-Dependent Localization of Clathrin Heavy Chain 1 During Meiosis

**FIGURE 2:**
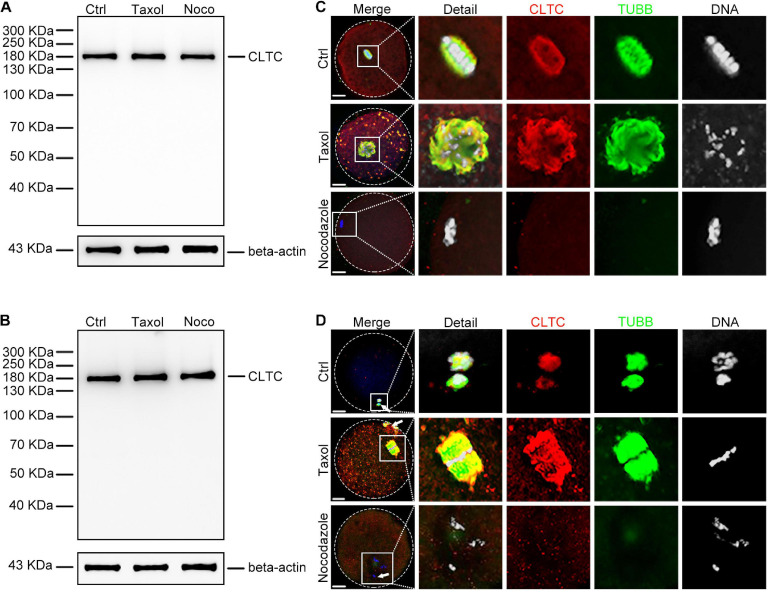
Expression and localization of CLTC in metaphase oocytes treated with either taxol or nocodazole. **(A)** Immunoblotting analysis of CLTC from MI-stage oocytes after treatment with taxol or nocodazole. Beta-actin was used as a loading control. Noco, nocodazole. **(B)** Immunoblotting analysis of CLTC from MII-stage oocytes after treatment with either taxol or nocodazole. Beta-actin was used as a loading control. Noco, nocodazole. **(C)** Subcellular localizations of CLTC in MI-stage oocytes after treatment with either taxol or nocodazole were detected by immunofluorescence staining, respectively. Slides were examined using a confocal microscope. Red, CLTC; green, TUBB; blue, DNA. Scale bar = 20 μm. **(D)** Subcellular localizations of CLTC in MII-stage oocytes after treatment with either taxol or nocodazole were detected by immunofluorescence staining, respectively. Slides were examined using a confocal microscope. White arrows indicate polar bodies. Red, CLTC; green, TUBB; blue, DNA. Scale bar = 20 μm. CLTC, clathrin heavy chain 1; MI, metaphase I; MII, metaphase II.

To further investigate the correlation between CLTC and MTs, we used the spindle-perturbing drugs taxol and nocodazole to treat sheep oocytes at both MI and MII stages. The immunoblotting results showed that taxol and nocodazole did not affect the amount of CLTC at the MI (Figure 2A) or MII stage (Figure 2B). However, analysis of the immunofluorescence staining revealed that MTs were overpolymerized and formed larger spindles after taxol treatment, and many asters appeared in the cytoplasm of both MI- (Figure 2C) and MII-stage oocytes (Figure 2D). Similar distribution and localization of CLTC were also seen in the cytoplasm (Figures 2C,D). In contrast, when sheep MI and MII oocytes were treated with nocodazole to induce MT depolymerization, the immunofluorescence results showed completely depolymerized MTs. In addition, CLTC localization was also dispersed within the cytoplasm (Figures 2C,D). These results suggested that CLTC is involved in MT depolymerization and polymerization.

**FIGURE 3:**
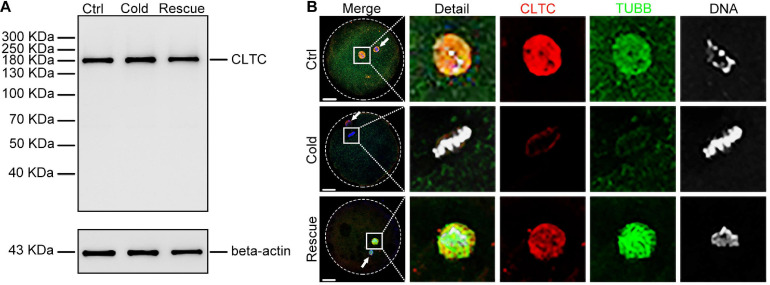
Expression and localization of CLTC in MII oocytes after cold treatment. **(A)** Immunoblotting analysis of CLTC from MII-stage oocytes after cold or rescue treatment. Beta-actin was used as a loading control. **(B)** Subcellular localizations of CLTC in MII-stage oocytes after cold or rescue treatment were detected by immunofluorescence staining. Slides were examined using a confocal microscope. White arrows indicate polar bodies. Red, CLTC; green, TUBB; blue, DNA. Scale bar = 20 μm. CLTC, clathrin heavy chain 1; MII, metaphase II.

The cold-mediated MT depolymerization assay was used to assess the relationship between CLTC and MTs. Sheep oocytes were placed in a cold environment (4°C) to depolymerize MII-stage MTs and then re-incubated for 30 min at 38.5°C. Immunoblotting results showed that the expression level of CLTC did not change after the cold and rescue treatments (Figure 3A). Immunofluorescence staining showed depolymerized MTs and dispersed CLTC in the cytoplasm after cold treatment (Figure 3B). After rescue treatment, depolymerized MTs reassembled into spindles and CLTC localization also appeared in spindle regions (Figure 3B). Thus, the subcellular localization of CLTC in sheep oocytes requires the involvement of the spindle in MII-stage oocytes.

### Knockdown of Clathrin Heavy Chain 1 Affects Spindle Assembly and Chromosome Congression During Oocyte Maturation

**FIGURE 4:**
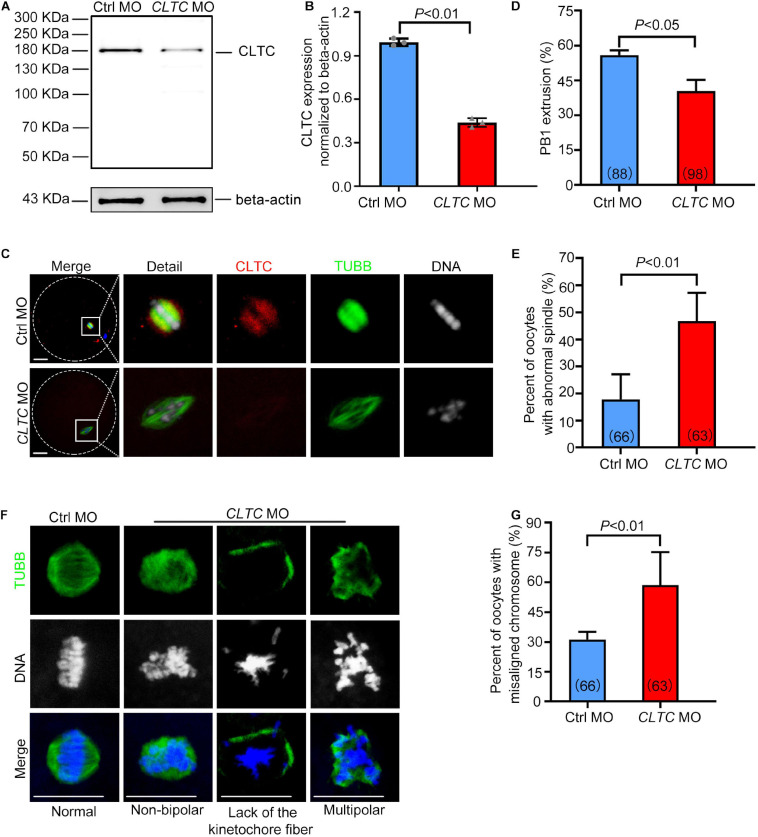
CLTC knockdown in sheep oocytes induces spindle defects and misaligned chromosomes. **(A)** Immunoblotting of CLTC expression after either *CLTC*-MO or control-MO injections. Beta-actin was used as a loading control. **(B)** Band intensities (representing CLTC expression) were assessed using gray-level analyses after *CLTC*-MO or control-MO injections. **(C)** The CLTC, spindles, and DNA in MII-stage oocytes (control-MO and *CLTC*-MO groups) were stained with anti-CLTC (red), anti-tubulin (green), and DAPI (blue), respectively. Scale bar = 20 μm. **(D)** The percentage of oocytes demonstrating PB1 extrusion in the control-MO and *CLTC*-MO groups. **(E)** Percentages of oocytes with abnormal spindles in the control-MO and *CLTC*-MO groups. **(F)** Immunofluorescence staining of CLTC expression after *CLTC*-MO or control-MO injections. Green, TUBB; blue, DNA. Scale bar = 20 μm. **(G)** Percentages of oocytes with chromosome misalignments in the control-MO and *CLTC*-MO groups. Data are presented as mean ± SD from at least three independent experiments. *P* < 0.05 was considered statistically significant. CLTC, clathrin heavy chain 1; MO, morpholino; PB1, first polar body; DAPI, 4′,6-diamidino-2-phenylindole.

To investigate the role of CLTC in sheep oocyte maturation, we microinjected *CLTC*-MO into sheep GV oocytes to knockdown CLTC expression. The immunoblotting results demonstrated that CLTC expression decreased by 56% in the *CLTC*-MO-injected group compared with the control group (Figures 4A,B). Consistently, the immunostaining showed that CLTC staining intensity was much lower in the *CLTC*-MO group compared with the control group (Figure 4C), demonstrating the efficiency of CLTC knockdown by MO injection. Compared with the control group, the percentage of first polar body (PB1)-extrusion oocytes in the *CLTC*-MO group was significantly lower (*P* < 0.05) (Figure 4D). Moreover, severe spindle-assembly defects were also observed in the *CLTC*-MO group (Figures 4E,F). These spindle-assembly defects were categorized into three main types: type I failed to form spindle poles; type II exhibited residual beta-tubulin at the poles without kinetochore fibers; and type III could form a spindle, but it was multipolar (Figure 4F). The percentage of oocytes with abnormal spindles was much higher in the *CLTC*-MO group (*P* < 0.01) (Figure 4E). In addition, chromosome-congression defects (misaligned chromosomes) were also observed in the *CLTC*-MO group (Figures 4F,G), and the incidence misalignment was much higher after CLTC knockdown (*P* < 0.01) (Figure 4G). These results indicated that CLTC knockdown affected both spindle formation and chromosome congression during oocyte meiosis progression.

### Knockdown of Clathrin Heavy Chain 1 Impairs Early Embryo Development in Sheep

**FIGURE 5:**
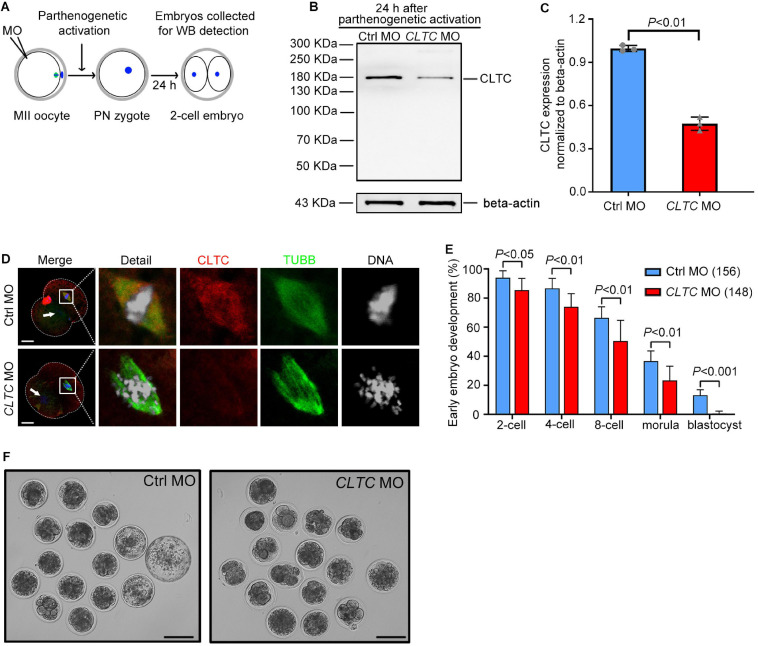
MII-stage knockdown of CLTC impairs embryonic development. **(A)** Scheme for sample collections for immunoblotting analyses after MO injections into MII-stage oocytes. **(B)** CLTC-expression immunoblotting after *CLTC*-MO or control-MO injections into MII-stage sheep oocytes. Beta-actin was used as a loading control. **(C)** Band intensities (representing CLTC expression) were assessed using gray-level analyses after *CLTC*-MO or control-MO injections in MII-stage sheep oocytes. Data are presented as mean ± SD from three independent experiments. *P* < 0.05 was considered statistically significant. **(D)** The CLTC, spindles, and DNA in two-cell embryos from the control-MO and *CLTC*-MO groups were stained with anti-CLTC (red), anti-tubulin (green), and DAPI (blue), respectively. Scale bar = 20 μm. White arrows indicate the other blastomere nucleus. **(E)** MII-stage sheep oocytes were injected with either *CLTC*-MO or control-MO, and then the rate of early embryo developmental was measured. Data are presented as mean ± SD from eight independent repeats, and at least 12 MII oocytes were included for parthenogenetic activation in each group for each repeat. *P* < 0.05 was considered statistically significant. **(F)** Representative images of early embryos after *CLTC*-MO or control-MO injections. Scale bar = 100 μm. MII, metaphase II; CLTC, clathrin heavy chain 1; MO, morpholino; DAPI, 4′,6-diamidino-2-phenylindole.

To explore the functional role of CLTC in early embryo development in sheep, *CLTC*-MO was microinjected into sheep MII oocytes. The oocytes were then activated by parthenogenesis and cultured. We assessed the developmental rates of early embryos from the two-cell stage to the blastocyst stage. Embryos cultured for 24 h after parthenogenetic activation (during which *CLTC*-MO had an inhibitory effect) were collected for immunoblot analysis (Figure 5A). The immunoblot assessment showed that CLTC expression was significantly impaired by the specific MO injection (Figures 5B,C). In addition, immunofluorescence detection of CLTC in two-cell-stage embryos that undergoing cell division showed diminished staining in the CLTC-knockdown group. Consistently, chromosome alignment was also disturbed after CLTC impairment, illustrated by failures of metaphase plate formation (Figure 5D). In order to determine the effects of CLTC knockdown on the developmental procedure of both zygotic to embryo transition and the later embryo development, the number of MII-stage oocytes instead of two-cell was used as denominator to calculate the percent of embryo development to each stage. Unsurprisingly, disturbing CLTC function before the two-cell stage significantly reduced the formation of two-cell embryos (*P* < 0.05), four-cell embryos (*P* < 0.01), eight-cell embryos (*P* < 0.01), and morula embryos (*P* < 0.01) (Figure 5E). Approximately 13% of embryos in the control group were able to develop into blastocysts, whereas almost all of the embryos in the *CLTC*-MO group were arrested at various developmental stages; none of them progressed past the morula stage. After 5 days of culture, the blastocyst rate in the *CLTC*-MO group was significantly lower compared with the control group (*P* < 0.001) (Figures 5E,F). These results indicated that CLTC played a crucial role in the early embryo development of sheep.

## Discussion

Here, we assessed the expression profile of CLTC during oocyte maturation and early parthenogenetic embryo development in sheep. We also assessed the relationship between TUBB and CLTC, the functional role of CLTC in spindle formation and cell division, and the influence of spindle-perturbing drugs, cold treatment, and expression knockdown. Our results have shown that a lack of CLTC caused lower PB1 extrusion, spindle assembly defects, misaligned chromosomes, and impaired early embryo development, and we conclude that CLTC plays a vital role in spindle formation and chromosome congression to regulate the progression of oocyte meiosis and early embryo mitosis in sheep.

Clathrin is a cytosolic protein that mediates endocytosis through vesicular transport during interphase and has a vital role in stabilizing MT and chromosomal alignment during mitosis (Kirchhausen, 2000; Holzenspies et al., 2010; Fielding and Royle, 2013). Our previous studies in the mouse showed that CLTC played a key role in meiotic maturation by stabilizing metaphase spindle and chromosome congression through cooperation with CKAP5 (Zhao et al., 2013; Lu et al., 2017). The present study is the first to report the necessity for CLTC during oocyte meiotic maturation and preimplantation embryo development in sheep. The oocyte expression of CLTC was maintained at a high level from the GV stage to the MII stage in sheep, which differs from our previous mouse results that CLTC expression was low at the GV stage and gradually increased to finally reach its highest level at the MII stage during oocyte meiotic maturation (Zhao et al., 2013). Oocyte maturation time in sheep required 22–24 h, considerably longer than the time required in mice (Wang et al., 2018, 2019); and as a result, more CLTC may be needed to regulate chromosomal separation during the longer maturation progress in sheep. In MII sheep oocytes, CLTC localized to the spindle, consistent with the results of our previous mouse study. The similar localization patterns of CLTC to the metaphase spindle areas of porcine, mouse, and sheep oocytes (Holzenspies et al., 2010; Zhao et al., 2013) indicate its fundamental functions in the spindle apparatus during oocyte meiosis. However, the colocalization between CLTC and tubulin appears to be only partial, with prominent extrusions at MI-stage spindle poles, which is different from our previous results (Zhao et al., 2013). In addition to diffuse cytoplasmic staining, we also found focal accumulations of CLTC at the plasma membrane in the interphase two-cell and four-cell sheep embryos. This is probably due to CLTC participating in reassembly of the Golgi apparatus near the plasma membrane (Radulescu et al., 2007) and is required for postmitotic Golgi reformation (Radulescu and Shields, 2012). Additional studies need to be carried out to determine any differences in CLTC localization between mice and sheep during oocyte maturation and early embryo development.

The taxol, nocodazole, and cold-treatment experiments were designed to explore the relationship between CLTC and TUBB. Taxol is frequently used in MT-dynamics research because it stabilizes MTs as well as promotes non-spindle MT formations (Cao et al., 2018). Here, we observed that taxol treatment caused CLTC to form many cytoplasmic asters with MTs. It has been reported that the TACC3/ch-TOG/clathrin complex can stabilize the MTs of the mitotic spindle as inter-MT bridges and that ch-TOG is vital to MT aster assembly after taxol treatment (Dionne et al., 2000; Booth et al., 2011). The present results confirm that CLTC is involved in regulating MT assembly in sheep oocytes. After nocodazole treatment, CLTC localization was evenly dispersed in the cytoplasm with the collapse of the MI- and MII-stage spindles. However, CLTC expression was almost unchanged as assessed by immunoblotting, and similar results were seen in mouse oocytes (Zhao et al., 2013). Cold treatment has also been shown to accelerate MT depolymerization, and the results are similar to those of nocodazole treatment (Rieder, 1981). Interestingly, the depolymerized MTs reassembled into spindles, and CLTC localized to the spindle regions following rescue treatment. We therefore speculate that CLTC may participate in the regulation of MT assembly and that the subcellular localization of CLTC to spindles is dependent on intact MTs. The drug- and cold-treatment results provide further support for the role of CLTC in spindle and MT assembly in meiotic oocytes from sheep.

In order to further explore the functions of CLTC in sheep oocytes, CLTC expression was knocked down by a specific MO oligomer. This knockdown indicated that CLTC is indispensable for meiotic spindle assembly due to the larger proportion of abnormal spindles and a decreased polar body extrusion rate in the *CLTC*-MO group compared with the control group. We further discovered that chromosomes were not aligned properly at the spindle equator, and it is possible that CLTC in sheep, as previously demonstrated in mouse (Zhao et al., 2013), also assists with this crucial spindle process for correct chromosomal alignment at metaphase. Clathrin is a triskelion consisting of three heavy chains (each with an associated light chain), and the N-terminal domain of each leg can bind to the mitotic spindle (Halebian et al., 2017). A bridge hypothesis has been proposed whereby clathrin acts as a brace between two or three MTs within a kinetochore fiber to increase fiber stability during mitosis (Kirchhausen and Harrison, 1981; Royle et al., 2005; Royle and Lagnado, 2006). Clathrin recruits phosphorylated TACC3 and ch-TOG to form an inter-MT bridge (the TACC3/ch-TOG/clathrin complex) to stabilize kinetochore fibers and ensure proper spindle assembly and chromosome alignment (Royle et al., 2005; Booth et al., 2011). The removal of TACC3–ch-TOG–clathrin led to a decrease in spindle length and significant alterations in kinetochore dynamics (Cheeseman et al., 2013), and additional research (Ma et al., 2013) has shown that functional interactions between SNX9 and CLTC also stabilize mitotic-spindle kinetochore (K)-fibers to ensure both chromosomal alignment and segregation. The present results of CLTC knockdown in meiotic sheep oocytes are similar to those described for mouse oocytes and indicate that CLTC plays a functional role to regulate spindle formation and chromosome congression in oocyte meiotic maturation together with CKAP5 (Zhao et al., 2013; Lu et al., 2017). Accordingly, the significant PB1 reduction seen in the *CLTC*-MO group may have been to spindle assembly defects and abnormal chromosome separation caused by CLTC knockdown, suggesting its importance during oocyte maturation in sheep as well.

To further explore the functional role of CLTC in sheep early embryo development, *CLTC*-MO was microinjected into MII-stage sheep oocytes before parthenogenesis activation and being cultured to different embryonic stages. Similarly, *CLTC-MO* can be applied to sheep embryos for functional disturbance. Consistently, we observed disturbed chromosomal alignments and failures in metaphase-plate formations in 2-cell-stage embryos. We further found that early embryo development was impaired after CLTC knockdown in MII oocytes resulting in almost all of the *CLTC*-MO embryos showing arrested development and lower developmental rates compared with the control-MO group. Based on previous centrosome-positioning research showing that clathrin negatively regulated the pulling forces acting on centrosomes and spindle poles in one-cell *Caenorhabditis elegans* embryos and maintained proper tension of the acto-myosin cortex (Spiro et al., 2014), we speculate that the organization and tension of the actin–myosin cortex was also impaired by CLTC depletion during mitosis in sheep early embryo development, resulting in arrested maturation. Other studies have shown thicker metaphase plates in clathrin-depleted NRK cells and that centromeres did not organize on the mitotic spindle in an orderly manner (Royle et al., 2005). Consistent with our present observation of chromosomal scatter, clathrin knockdown was also shown to increase the incidence of misaligned chromosomes in metaphase-like cells and to destabilize kinetochore fibers, which led to defective chromosomal congression at the metaphase plate. Accordingly, we suggest that the CLTC regulatory mechanism in cell division at early embryo stages is similar to that for somatic mitotic cell division. Therefore, CLTC appears to be essential for early embryo development in sheep, but detailed mechanisms for its regulation of early embryo development remain to be elucidated.

In the present study, we have documented CLTC expression and localization during oocyte meiotic maturation and early embryo development in sheep. Taxol, nocodazole, and cold treatments were used to reveal the relationship between CLTC and TUBB. CLTC knockdown disrupted sheep oocyte meiotic spindle assembly and chromosomal alignment and impaired embryo-development potential. In conclusion, the results demonstrate that CLTC plays a vital role in regulating spindle assembly, chromosome congression during oocyte meiotic maturation, and early embryo developmental progression in sheep. In addition, this is one of few studies addressing the role of MT-associated proteins in oocytes and early embryo development in domesticated animals, providing additional fundamental knowledge related to their reproduction.

## Data Availability Statement

The raw data supporting the conclusions of this article will be made available by the authors, without undue reservation.

## Ethics Statement

The animal study was reviewed and approved by the Ethics Committee of Inner Mongolia University (Approval number: SYXK 2014-0002).

## Author Contributions

C-GL, D-JZ, ZH, XH, C-JZ, and JW conceived and designed the experiments and analyzed the data. ZH, XH, C-JZ, JW, XW, X-YW, D-JZ, and C-GL performed the experiments. ZH, XH, C-JZ, JW, and C-GL wrote the manuscript. All authors contributed to the article and approved the submitted version.

## Conflict of Interest

The authors declare that the research was conducted in the absence of any commercial or financial relationships that could be construed as a potential conflict of interest.
